# Supplementary material for: Cortisol levels after cold exposure are independent of adrenocorticotropic hormone stimulation
Source: PLoS One. 2020 Feb 18;15(2):e0218910. doi: 10.1371/journal.pone.0218910 (PMC7028257; doi:10.1371/journal.pone.0218910)
Supplement: S1 Fig — Relationship between the cortisol level in blood collected at different sites and sex (a), age (b), survival period (c), and postmortem period (d) in all cases. The difference in males and females was associated with the difference in number. However, in terms of age, survival period, and postmortem period, there was a correlation, so the number did not affect the cortisol level. There was no difference between males and females in terms of collection site. (PPTX) [file pone.0218910.s001.pptx]

## Slide 1
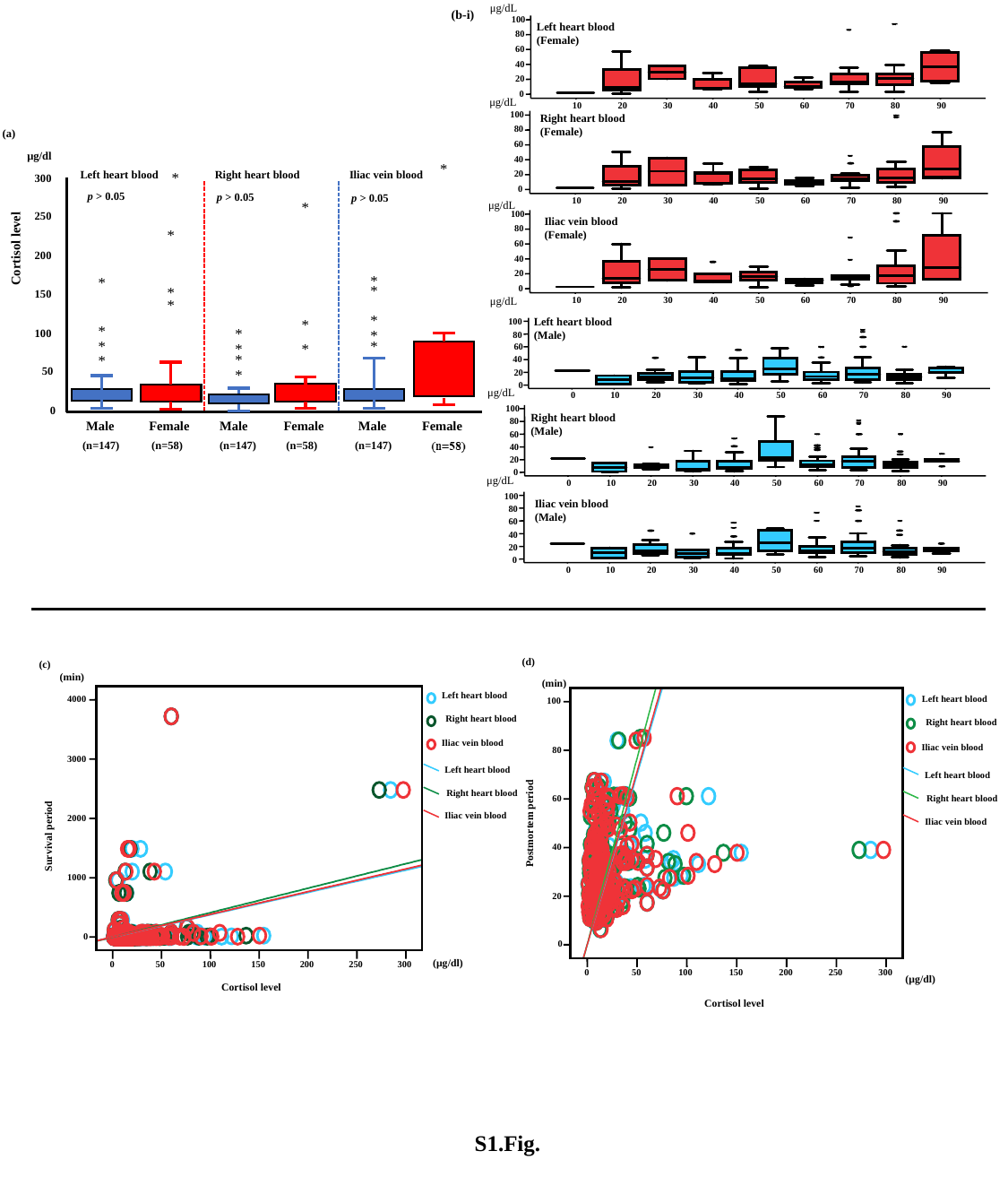

(b-i)
μg/dL
100
80
60
40
20
0
10
20
30
40
50
60
70
80
90
Right heart blood
(Female)
100
80
60
40
20
0
10
20
30
40
50
60
70
80
90
100
Iliac vein blood
(Female)
80
60
40
20
0
10
20
30
40
50
60
70
80
90
Left heart blood
(Male)
100
80
60
40
20
0
0
10
20
30
40
50
60
70
80
90
100
Right heart blood
(Male)
80
60
40
20
0
0
10
20
30
40
50
60
70
80
90
Iliac vein blood
(Male)
100
80
60
40
20
0
0
10
20
30
40
50
60
70
80
90
Left heart blood (Female)
μg/dL
(a)
μg/dl
*
Left heart blood
Iliac vein blood
Right heart blood
*
300
p > 0.05
p > 0.05
p > 0.05
*
μg/dL
250
*
Cortisol level
200
*
*
*
*
150
μg/dL
*
*
*
*
*
100
*
*
*
*
*
*
*
50
*
μg/dL
0
Male
Female
Male
Female
Male
Female
(n=147)
(n=58)
(n=147)
(n=58)
(n=147)
μg/dL
(d)
(c)
(min)
(min)
Left heart blood
Left heart blood
4000
100
Right heart blood
Right heart blood
Iliac vein blood
Iliac vein blood
80
3000
Left heart blood
Left heart blood
Right heart blood
60
Right heart blood
Survival period
Postmortem period
Iliac vein blood
2000
Iliac vein blood
40
1000
20
0
0
(μg/dl)
0
50
100
150
200
250
300
(μg/dl)
0
50
100
150
200
250
300
Cortisol level
Cortisol level
S1.Fig.
