# Supplementary material for: Cortisol levels after cold exposure are independent of adrenocorticotropic hormone stimulation
Source: PLoS One. 2020 Feb 18;15(2):e0218910. doi: 10.1371/journal.pone.0218910 (PMC7028257; doi:10.1371/journal.pone.0218910)
Supplement: S2 Fig — Left and right cardiac blood (a), left cardiac blood-iliac vein blood (b), and right cardiac blood-iliac vein blood (c). (PPTX) [file pone.0218910.s002.pptx]

## Slide 1
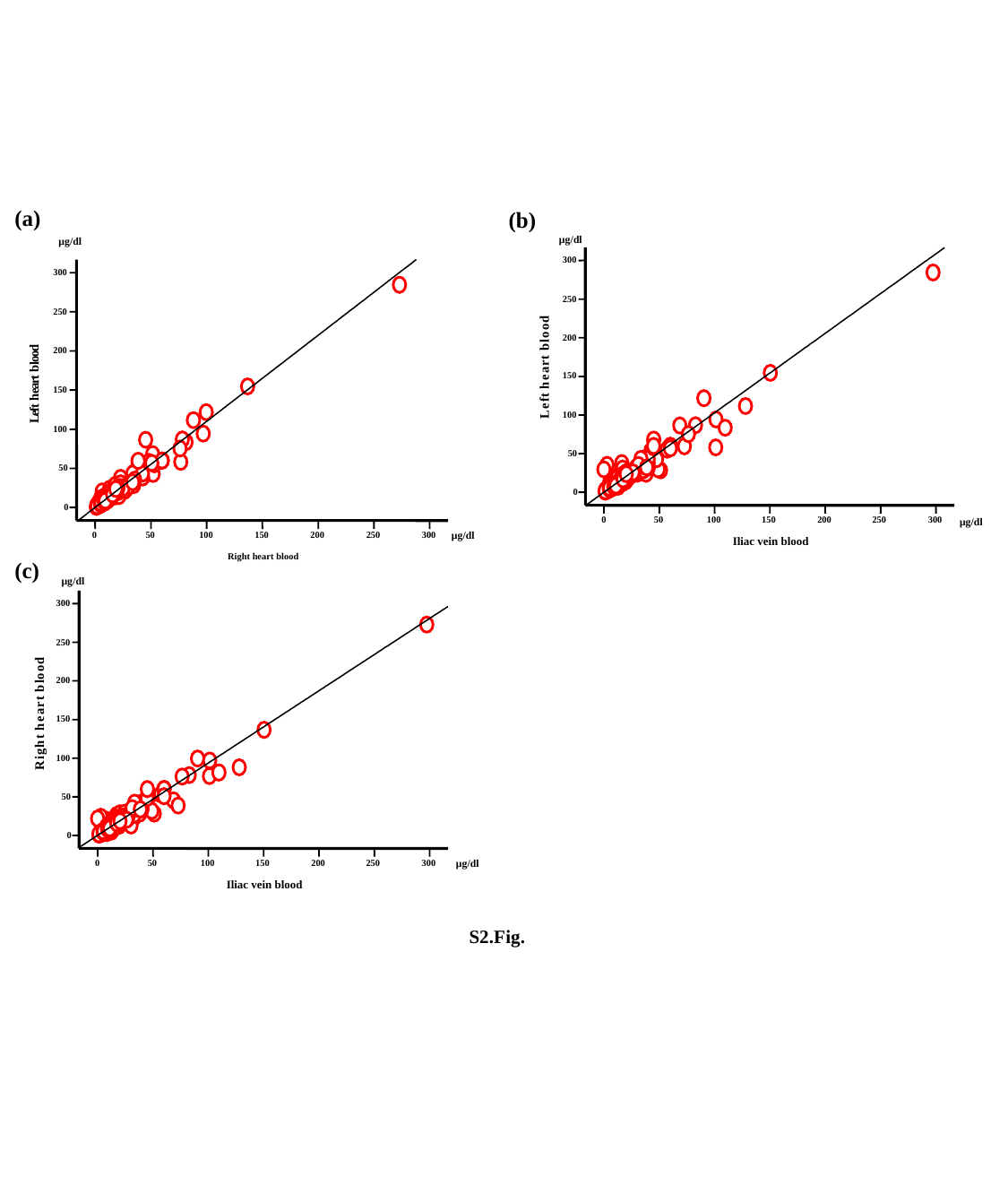

(a)
(b)
μg/dl
μg/dl
300
300
250
250
d
o
o
200
l
b
d
200
o
t
o
r
l
b
a
t
e
150
r
h
a
e
150
t
h
f
t
e
f
e
L
100
L
100
50
50
0
0
μg/dl
0
50
100
150
200
250
300
μg/dl
0
50
100
150
200
250
300
Iliac vein blood
(c)
Right heart blood
μg/dl
300
250
d
o
o
l
200
b
t
r
a
e
150
h
t
h
g
i
100
R
50
0
μg/dl
0
50
100
150
200
250
300
Iliac vein blood
S2.Fig.
